# Relationship between some indicators of reproductive history, body fatness and the menopausal transition in Hungarian women

**DOI:** 10.1186/s40101-015-0076-0

**Published:** 2015-10-22

**Authors:** Annamaria Zsakai, Nicholas Mascie-Taylor, Eva B Bodzsar

**Affiliations:** Department of Biological Anthropology, Eotvos Lorand University, Pazmany P. s. 1/c, 1117 Budapest, Hungary; Department of Biological Anthropology, University of Cambridge, Pembroke Street, Cambridge, CB2 3RA UK

**Keywords:** Menopause, Age at menopause, Parameters of menstrual and reproductive history, Obesity, Survival analysis

## Abstract

**Background:**

This paper analyzed the relationship between some indicators of reproductive history and body fatness in relation to the timing of the menopause transition in Hungarian women using survival analysis after controlling for birth cohort.

**Methods:**

Data on menstruation and reproductive history were collected during the personal interviews in a sample of 1932 women (aged 35+ years). Menarcheal age, the length of menstrual cycles and menstrual bleedings, regularity of menstrual cycles, number of gestations, lactation, the ever use of contraceptives, menopausal status and age at menopause were used as indicators of reproductive history. The body fat fraction was estimated by bioelectrical impedance analysis. Body fatness was also estimated by dividing women into obese and non-obese categories (considering body mass index and waist-to-hip ratio). Survival analyses were used to analyze the relationship between the indicators of reproductive history and body fatness during the menopausal transition.

**Results:**

Only the menarcheal age among the investigated reproductive life characteristics showed secular changes in the studied decades in Hungary; the mean age at menarche decreased by approximately 2.5 months per decade from the 1920s until the 1970s. Ever use of hormonal contraceptives, a relatively long cycle length in the perimenopausal transition and higher parity were all related with lower risk of early menopause. Later menarcheal age, normal length of menstrual cycle or bleeding in the climacterium, irregular bleeding pattern and postmenopausal status were associated with a higher amount of body fatness, while never use of contraceptives, regular menstruation, postmenopausal status and relatively early menopause were associated with a higher risk of abdominal obesity.

**Conclusion:**

This report confirms that age of menarche is not significantly predictive of age at menopause but prior use of oral contraceptives, longer mean cycle length and smaller number of gestations all are. In addition, age of menarche, irregular bleeding pattern before the climacterium, length of menstrual cycles and bleedings during the climacterium and postmenopausal status were associated with obesity during the climacterium.

## Background

The age at menopause is a distinctive milestone in female reproductive life since it indicates not only the increased risk of morbidity and premature mortality due to the decreased level of sex hormone levels but also a biological marker of overall ageing and general health status. Menopause is universal and shows little variation in the timing of its occurrence (~50 years) across contemporary human populations and has remained mainly constant over the last 100 years in developed societies [1–3].

Previous studies on the onset of natural menopause have revealed that its variance is determined mainly by the interactions of multiple genes through control of the ageing processes of the neuroendocrine system [4, 5], and environmental factors explain only a small part of the variance in the age of menopause. It has been found that lifestyle factors and general health status can influence reproductive ageing by damaging the oocytes and decreasing the level of sexual hormones [6, 7].

The main purposes of this study were to analyze (1) the relationship between the age at menopause and the other characteristics of reproductive life in Hungarian women and (2) the relationships between body fatness at the time of menopause and reproductive life characteristics. The secular changes of the studied reproductive variables were analyzed only in those women who never used hormonal contraceptives, since the use of this kind of contraceptives can significantly modify the natural characteristics of menstrual cycles.

By considering the main purposes of the study and the results of the former menopause studies, the following hypotheses were posed:

1. Since it was confirmed by many epidemiological surveys [8] that the improvement of the socioeconomic environment in the last century significantly influenced the pattern of growth and maturation (the block of the growth velocity was disengaged, the manifestation of genetically established metric features increased, the sexual maturation shifted toward an earlier chronological age), the positive secular trend of menarcheal age was hypothesized.
2. According to our hypothesis, the age at menopause is influenced not only by extrinsic factors (e.g. by socioeconomic factors) but also by the factors of the reproductive life; therefore, the relationship between the age at menopause and the other main characteristics of reproductive life (as menarcheal age, number of gestations, lactation, length of menstruation cycles) was presupposed.
3. The possible advancement in timing of puberty was hypothesized to be accompanied by the earlier onset of menopause due to the positive secular socioeconomic changes and the earlier onset of reproductive life and a decrease in the number of gestations per women over recent decades.
4. Since the transitions between prereproductive, reproductive and postreproductive stages are accompanied not only by the changing levels of female sex hormones but also by significant changes of metabolic status and body structure, a hypothesis of a relation between the risk of obesity/abdominal obesity and the characteristics of reproductive life was proposed as well. The characteristics of reproductive life that can be related to higher level of female sex hormones (e.g. earlier menarcheal age, oral contraceptives) were supposed to increase the risk of obesity in the menopausal transition because these reproductive life parameters were considered as indicators of a lifelong higher level of sex hormones.

## Methods

### The sample

Subject recruitment was done by using multilevel multistage sampling so as to obtain subjects that represented the diverse types of settlements (types of settlements were as follows: Budapest with two million inhabitants, large towns with more than 100,000 inhabitants, small towns with 10,000−99,999 inhabitants, villages with 1000−9999 inhabitants, small villages below 1000 inhabitants) as well as the age distribution of Hungarian women [9]. The sample was designed by the Hungarian Central Statistical Office. Women living in the selected settlements were contacted (via personal interviews at home or place of residence and workplaces) and were screened for eligibility (eligibility criteria were as follows: age between 25 and 94 years, able to give verbal consent; elderly people under guardianship was excluded). This process continued until a sufficient number of eligible women was identified in all settlement categories in every age-group. The socioeconomic status of the sample (by considering the level of subjects’ education and the marital status: 8.5 % not completed elementary school, 22.4 % completed elementary school, 16.7 % vocational training school, 35.3 % secondary school, 17.1 % higher education; 12.4 % never married, 41.0 % married, 28.5 % widowed, 18.1 % divorced or separated) fitted the socioeconomic stratification of the selected age-group of women in the Hungarian population [9]. Participation was voluntary and data were anonymised and analyzed for scientific purposes only.

The main purposes of the Hungarian Menopause Study were (1) to analyze the nature of menopausal transition in Hungary; (2) to analyze the manner in which the onset of menopause and the length of menopausal transition are affected by the nutritional status and body composition, lifestyle and socioeconomic background of women; (3) to estimate the influence of genetic and endocrinological factors on reproductive ageing process by comparing autophagic activity and the female sex hormone levels in women being in different menopausal status; and (4) to identify the most important intrinsic and extrinsic risk factors that can lead to early or late menopause.

Study volunteers included 1932 Hungarian women aged between 25 and 94 years (mean ± SD of age, 64.79 ± 16.33 years, see Table 1) who were enrolled in this cross-sectional research during 2011–2014 (the Hungarian Menopause Study was done by the research team of the Department of Biological Anthropology, Eotvos Lorand University in the work places, community centres, nursing homes of the subjects and in the department as well). Multiple anthropometric measurements, including body mass, stature, biacromial width, chest width and depth, biepicondylar humeral and femoral widths, circumferences of relaxed upper arm, lower arm, wrist, hand, waist, hip, calf and ankle and skinfolds at the biceps, triceps, subscapular, suprailiac and calf locations, were assessed following standard protocols and methods (IBP recommendations) [10, 11].

**Table 1:** The distribution of studied women (born between the 1920s and 1980s) by birth cohort and menopausal status

Birth cohort	Number	Percentage	Menopausal status	Number	Percentage
1920 (1920–1929)	320	16.6	Premenopausal	475	31.2
1930 (1930–1939)	347	18.0	Early perimenopausal	53	3.5
1940 (1940–1949)	275	14.2	Late perimenopausal	51	3.4
1950 (1950–1959)	357	18.5	Postmenopausal	941	61.9
1960 (1960–1969)	256	13.3	Hyster- or ovariectomized	401	–
1970 (1970–1979)	192	9.9	Unknown	11	–
1980 (1980–1989)	185	9.6			
Total	1932	100.0	Total	1932	100.0

After anthropometric, body composition and osteometric examinations, women (the anthropometric description of women is presented in Table 2) were interviewed using pre-tested, interviewer-administered questionnaires concerning their reproductive and menstrual history, socio-demographic background, lifestyle (habitual physical activity and nutrition), morbidity and subjective health status (all measurements and interviews were obtained by highly experienced investigators).

**Table 2:** Anthropometric description of the studied sample (for continuous parameters—mean, SD, 95 % CI; for discrete parameters—relative frequency of the categories)

	Mean	SD	95 % CI	
Body mass (kg)	69.28	14.52	68.60	69.96
Stature (cm)	157.05	7.10	156.71	157.38
Upper arm circumference (cm)	27.52	3.79	27.34	27.70
Waist circumference (cm)	91.38	13.20	90.74	92.02
Hip circumference (cm)	104.22	12.47	103.62	104.83
Absolute body fat mass (kg)	23.24	9.88	22.78	23.71
Relative body fat mass (%)	32.34	8.40	31.84	32.64
BMI (kg/m^2^)	28.06	5.47	27.81	28.32
WHR (cm/cm)	0.876	0.056	0.873	0.878
Nutritional status categories	%			
Underweight	2.1			
Normal nutritional status	27.9			
Overweight	36.8			
Obese	33.2			
Abdominal obesity categories	%			
Not abdominal obese	31.1			
Abdominal obese	68.9			

*SD* standard deviation, *95 % CI* 95 % confidence interval

Women who had any diseases or were taking any medications known to affect body composition or who had been hysterectomized or bilaterally ovariectomized were not included in the present analysis. During the analysis of secular changes in the reproductive life variables, only the data of those women who had never used oral contraceptives were considered.

### Reproductive life characteristics

Data on menarcheal age, length of menstrual cycles and menstrual bleeding in puberty, youth, adulthood and climacterium, ever use of hormonal contraceptives (including past and current oral contraceptive use: date, length of use, name of contraceptives), number of pregnancies (maternal age at pregnancies, length of pregnancies), number of live births with or without lactation, age at which any irregularity of menstrual cycle length commenced, age at the last menstrual cycle and age at menopause were collected during the personal interviews. The questionnaires were constructed by considering the WHO and Stages of Reproductive Aging Workshop (STRAW) recommendations on collecting data on reproductive life characteristics in women [12, 13].

Subjects were divided into four groups of premenopausal, early and late perimenopausal and postmenopausal on the basis of their menstrual cycle characteristics by considering the WHO and STRAW recommendations [12, 13] (the occurrence of irregular periods and the age of last menstrual period; Table 3). The age of natural menopause was estimated by considering the age of the last menstrual cycles in the postmenopausal subgroup. Subjects were divided into 10-year birth cohorts (Table 1).

**Table 3:** Menstrual and reproductive history parameters of studied Hungarian women (*n* 1932, born between the 1920s and 1980s) included in multivariate survival analyses

Menstrual and reproductive variables	Definition of categories
Menarcheal age	
Relative early	≤10.9 years
Average	11.0–13.9 years
Relative late	≥14.0 years
Length of menstrual cycles	
Relatively short	≤27 days
Average	28–30 days till adulthood and 28–70 days in climacterium
Relatively long	≥31 days till adulthood and ≥71 days in climacterium
Length of menstrual bleeding	
Relatively short	≤3 days till adulthood and −2 days in climacterium
Average	4–6 days till adulthood and 3–6 days in climacterium
Relatively long	≥7 days
Number of gestations and number of gestations with or without lactation	1
	2
	3
	4 or more
Menopausal status	
Premenopausal	Menstrual period in the past 3 months, no decreased predictability
Early perimenopausal	Menstrual period in the past 3 months but less predictability in the preceding 12 months
Late perimenopausal	Menstrual bleeding in the past 12 months but not in the past 3 months
Postmenopausal	Amenorrheic for the past 12 months
Age at menopause	
Relative early	≤43.9 years
Average	44.0–53.9 years
Relative late	≥54.0 years

### Body fatness estimation

The body fat fraction was estimated by bioelectrical impedance analysis (NutriGuard M device, DataInput, Germany). Subjects were divided into nutritional status categories (underweight: BMI < 18.50 kg/m^2^, normal nutritional status: BMI = 18.50-24.99 kg/m^2^, preobse/overweight: 25.00-29.99 kg/m^2^, obese: BMI > 30.00 kg/m^2^) using the WHO cut-off points of BMI [14]. Waist-to-hip ratio was used to assess abdominal obesity. Subjects were divided into abdominal obesity categories by using WHO cut-off point (abdominal obesity: WHR ≥ 0.85) [15].

### Statistical analysis

As some women had not reached menopause, survival analyses (Kaplan-Meier and Cox proportional hazards regression models) were used to analyze the data. Initially, analyses were undertaken using only one independent variable (potential estimates of age at menopause were as follows: menarcheal age, the length of menstrual cycles and menstrual bleeding in puberty, youth, adulthood and climacterium, ever use of hormonal contraceptives, the number of pregnancies, lactation, birth cohort; potential estimates of obesity indicators were the same reproductive characteristics as in the case of age at menopause by adding menstrual irregularity, menopausal status and age at menopause) and those that were significant were entered into a multivariate analysis (stepwise analysis). The omnibus test was used for testing the overall fit of Cox regression models.

The statistical analyses were done by using SPSS v. 20. Hypotheses were tested at the 5 % level of random error.

### Ethics committees’ informed consent

All subjects were asked to give their written informed consent to participate in the study. All attendants were provided by detailed information on the main purposes of the study and on all examinations before their approval. The participation was voluntary and anonym in the study. The research objectives, the research methodology and the questionnaires were approved by the National Human Research Ethics Committee (108/2011) and the Hungarian Scientific Research Fund (EIK-1001/2011).

### Limitations of the study

The main limitations of the study were the cross-sectional study design and the retrospective data collection (the possibility of recall bias cannot be excluded, especially not among older respondents). The personal interviews helped to diminish this methodological limitation of the study. The menopausal status and the reproductive life characteristics, especially the characteristics of the menstrual cycles, can be estimated more accurately by longitudinally. Moreover, the menopausal status can be estimated more accurately by following the complete STRAW staging system for ovarian ageing including menstrual and qualitative hormonal criteria [13]. Although the STRAW system is widely considered as the gold standard for characterizing the menopausal transition, but due to the total cost of qualitative hormonal estimations, this system cannot be always used in epidemiological studies, mostly in clinical surveys. Therefore, only the characteristics of menstrual cycle pattern were considered to divide women into menopausal status subgroups in the present analysis.

## Results

### Secular changes in the characteristics of reproductive life

**Fig. 1:**
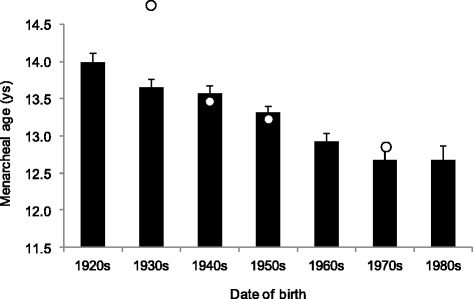
The mean (+SE) age of menarche (*p* < 0.001, ANOVA) by the birth cohorts of Hungarian women (○: status quo data collection, median values, cit by Bodzsar 1998)

Menarcheal age showed a significant positive secular change between the 1920s and 1980s: the mean age of menarche decreased by birth cohorts from the age of ~14.0 years to 12.7 years (Fig. 1, Table 4) in the youngest cohort. Due to observed secular change, all later regression analyses are adjusted for possible cohort effects.

**Table 4:** Statistical parameters (mean, SD, 95 % CI) of menarcheal age and age at menopause by birth cohort

	Mean	SD	95 % CI		Mean	SD	95 % CI	
1920–1929	14.00	1.82	13.76	14.23	51.78	5.19	50.92	52.75
1930–1939	13.61	1.77	13.46	13.86	51.22	5.49	50.45	52.02
1940–1949	13.57	1.68	13.34	13.80	52.03	4.86	51.06	53.12
1950–1959	13.31	1.34	13.14	13.49	51.25	5.21	50.65	51.93
1960–1969	12.93	1.39	12.72	13.13	49.33	3.75	48.43	50.25
1970–1979	12.68	1.64	12.34	12.98	–	–	–	–
1980–1989	12.67	1.53	12.30	13.03	–	–	–	–
Together	13.45	1.66	13.33	13.56	51.40	5.04	51.00	51.99

*SD* standard deviation, *95 % CI* 95 % confidence interval

**Fig. 2:**
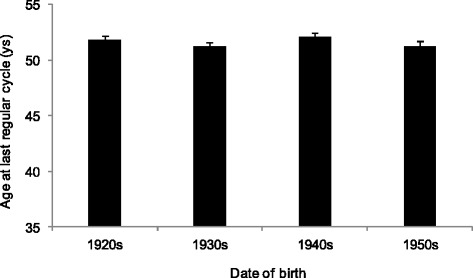
The mean (+SE) age at menopause by the birth cohorts of the studied Hungarian women (*p* = 0.545, ANOVA)

**Fig. 3:**
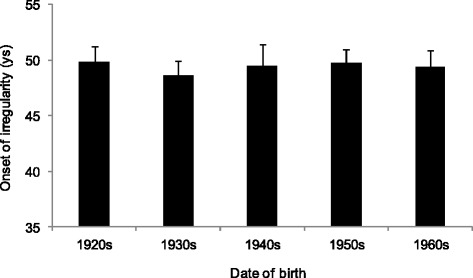
The mean (+SE) age of onset of bleeding irregularity by the birth cohorts of the studied Hungarian women (*p* = 0.852, ANOVA)

The age at menopause did not change in the birth cohorts, and the median age at menopause was 51.6 years in these Hungarian women (Fig. 2, Table 4). The onset of perimenopause (the onset of menstrual irregularity in the beginning of the menopausal transition) did not show any secular change, and its median age was 48.4 years, indicating that perimenopause started about 3 years before the onset of menopausal amenorrhea (Fig. 3).

**Fig. 4:**
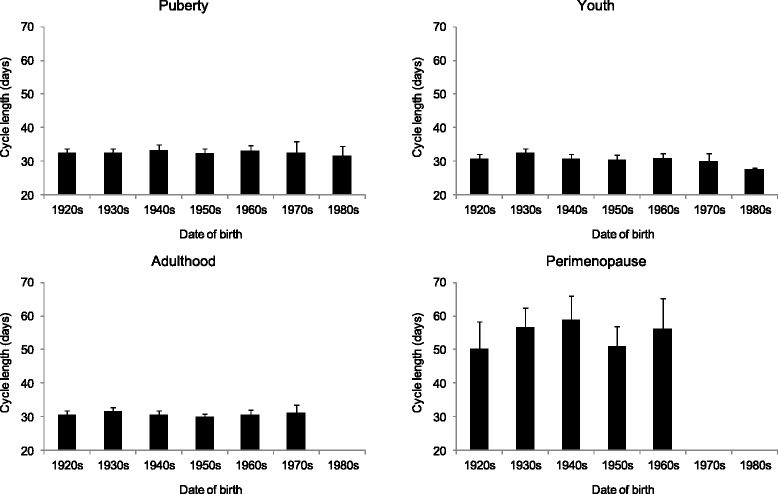
The mean (+SE) cycle length by the birth cohorts of the studied Hungarian women (puberty: *p* = 0.973, youth: *p* = 0.614, adulthood: *p* = 0.732, perimenopause: *p* = 0.759, ANOVA)

**Fig. 5:**
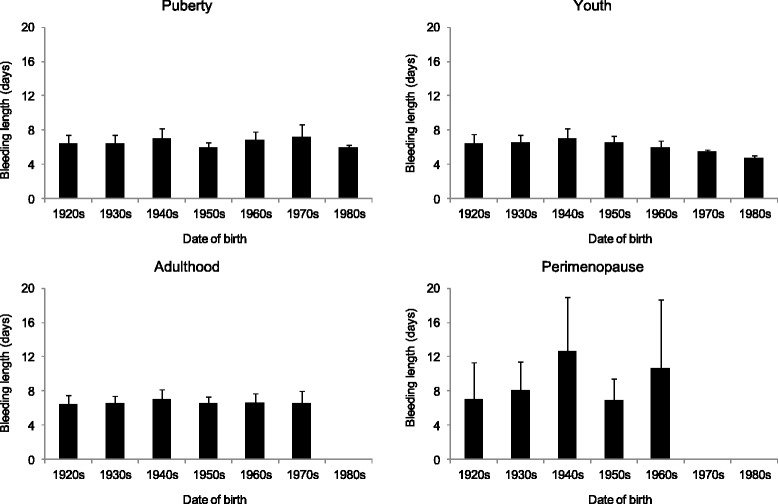
The mean (+SE) bleeding length by the birth cohorts of the studied Hungarian women (puberty: *p* = 0.968, youth: *p* = 0.960, adulthood: *p* = 0.908, perimenopause: *p* = 0.461, ANOVA)

By considering the length of menstrual cycles in the birth cohorts of women who never used oral contraceptives, it was found that the menstrual cycle length did not change significantly during the studied decades in either of the life cycle stages. Length of the menstrual cycle was stable until adulthood, ranging from 31 to 32 days. However, a significant increase occurred between adulthood and climacterium with cycle lengths of 54–55 days. Furthermore, an increase in the variability of cycle length accompanied this increase toward the perimenopause status (Fig. 4). The length of menstrual bleeding (i.e. the menstrual phase in the cycles) did not show any secular change. This characteristic of menstrual cycle pattern was stable until the perimenopause period, but a significant increase both in the length and the variability of bleeding was found from adulthood toward the climacterium (Fig. 5).

### Reproductive life parameters and the onset of menopause

Ever use of hormonal contraceptives, the menstrual cycle length in the perimenopause, the number of gestations and the birth cohort were found to be associated with the risk of early menopause in the univariate analysis (Table 5). Multivariate Cox proportional hazards regression analyses confirmed associations of reproductive life parameters with earlier onset of menopause (Table 6). Specifically, ever use of contraceptives was significantly associated with ~50 % lower risk of earlier menopause. In addition, both relatively short and average cycle lengths during the perimenopausal transition increased risk of earlier menopause compared to relatively long cycles in the perimenopausal stage by 87 and 77 %, respectively. However, risk of early menopause decreased as number of gestations increased (Table 6).

**Table 5:** Significance levels (*p* (*χ* ^2^), significant values are in italics) in the Kaplan-Meier univariate survival analysis of the relation between the studied reproductive parameters and the age at menopause, body fatness and abdominal obesity in the studied Hungarian women (*n* 1932, born between the 1920s and 1980s)

Risk factors	Age at menopause	Body fatness	Abdominal obesity
Hormonal contraceptives	*0.037* (4.37)	0.594 (0.28)	*<0.001* (14.54)
Cycle length: puberty	0.961 (0.08)	0.569 (1.13)	0.308 (2.64)
Youth	0.420 (1.73)	0.919 (0.17)	0.664 (0.82)
Adulthood	0.246 (2.80)	0.895 (0.22)	0.547 (1.21)
Climacterium	*0.016* (4.36)	*0.081* (5.03)	0.335 (2.19)
Bleeding length: puberty	0.189 (3.33)	0.782 (0.49)	0.516 (1.32)
Youth	0.100 (4.60)	0.955 (0.09)	0.442 (1.63)
Adulthood	0.096 (4.69)	0.994 (0.01)	0.318 (2.29)
Climacterium	0.663 (0.82)	*0.087* (4.87)	0.481 (1.46)
Number of gestations	*0.028* (5.07)	0.292 (3.73)	0.422 (2.81)
Age of menarche	0.790 (0.47)	*0.030* (7.01)	0.790 (0.47)
Lactation	0.879 (0.02)	0.143 (2.14)	0.621 (0.24)
Birth cohorts	*<0.001* (49.83)	*<0.001* (95.40)	*<0.001* (43.75)
Menstrual irregularity	–	*0.029* (4.79)	*<0.001* (29.70)
Menopausal status	–	*0.003* (15.98)	*<0.001* (20.69)
Age at menopause	–	0.302 (2.39)	0.394 (1.86)

**Table 6:** Hazard ratio (HR) of the onset of menopause according to the studied reproductive parameters (adjusted for birth cohorts) in the Cox proportional regression modelling (95 % CI, *p* = 0.019, omnibus test)

Reproductive life variables		HR	95 % CI	
Hormonal contraceptives	No^a^	1.000	–	–
	Yes	0.522	0.271	1.005
Cycle length in climacterium	Short	1.868	0.771	4,525
	Average	1.767	1.009	3.097
	Long^a^	1.000	–	–
Number of gestations	0^a^	1.000	–	–
	1	0.868	0.219	2.038
	2	0.702	0.298	2.161
	3	0.661	0.187	1.971
	4+	0.697	0.273	2.156

*95 % CI* 95 % confidence interval

^a^Reference category in the analysis

### Body fatness in the menopause transition in relation to the reproductive history

By considering the relationship between the reproductive variables and fatness in the menopausal transition, it was found that relatively later age of menarche, normal length of menstrual cycle or bleeding in the climacterium, irregular bleeding pattern as well as the postmenopausal status were associated with a higher amount of body fatness, higher prevalence of obesity in the menopause transition (Tables 5 and 7). However, the never use of contraceptives, the regular menstruation, the postmenopausal status and relatively earlier menopausal age associated with a higher risk of abdominal obesity (Tables 5 and 8).

**Table 7:** Hazard ratio (HR) of obesity according to the studied reproductive parameters (adjusted for birth cohorts) in the Cox proportional regression modelling (95 % CI, *p* = 0.020, omnibus test)

Reproductive life variables		HR	95 % CI	
Menarcheal age	Early	0.694	0.285	2.687
	Normal	0.492	0.279	0.867
	Late^a^	1.000	–	–
Cycle length in climacterium	Short	0.790	0.361	1.729
	Average^a^	1.000	–	–
	Long	0.641	0.334	1.224
Bleeding length in climacterium	Short	0.335	0.134	0.836
	Average^a^	1.000	–	–
	Long	0.890	0.461	1.719
Menstrual irregularity	No	0.926	0.205	4.083
	Yes^a^	1.000	–	–
Menopausal status	Premenopausal	0.398	0.117	1.346
	Early perimenopausal	0.162	0.062	0.611
	Late perimenopausal	0.422	0.122	0.952
	Postmenopausal^a^	1.000	–	–

*95 % CI* 95 % confidence interval

^a^Reference category in the analysis

**Table 8:** Hazard ratio (HR) of higher abdominal body fat distribution (abdominal obesity) according to the studied reproductive parameters (adjusted for birth cohorts) in the Cox proportional regression modelling (95 % CI, *p* = 0.048, omnibus test

Reproductive life variables		HR	95 % CI	
Hormonal contraceptives	No	1.084	0.877	1.306
	Yes^a^	1.000	–	–
Menstrual irregularity	No	1.162	0.504	2.013
	Yes^a^	1.000	–	–
Menopausal status	Premenopausal	0.674	0.294	3.933
	Early perimenopausal	0.605	0.236	1.491
	Late perimenopausal	0.611	0.233	1.576
	Postmenopausal^a^	1.000	–	–
Age at menopause	Early	1.233	1.012	1.576
	Normal	1.142	0.897	1.513
	Late^a^	1.000	–	–

*95 % CI* 95 % confidence interval

^a^Reference category in the analysis

## Discussion

By considering the secular changes in the studied reproductive parameters, it could be stated that the results of presented retrospective data collection were in very good concordance with the results of status quo data collection on menarcheal age in the last century [16]; the menarcheal age decreased significantly in the last decades in Hungary. The observed menopausal age (median age 51.6 years) was stable in the studied decades and consistent with other available data from Europe with median age at menopause in this region ranging between 50 and 52 years. This means that the length of the reproductive period extended with about 1.5 year in the studied interval. Neither the length of the menstrual cycle nor the menstrual flow showed secular change in the studied decades.

Published reports are not in agreement with relationships among reproductive factors or with associations of reproductive variables with body fatness in the menopausal transition (Table 9). For example, the main risk factors of early menopause were identified as

**Table 9:** Reproductive risk factors of early menopause, obesity and abdominal obesity in the menopause transition—a review of literature (reviewed from the late 1990s)

Higher risk of early menopause	Higher risk of obesity in the climacterium	Higher risk of abdominal obesity in the climacterium
Late menarcheal age [17, 18]	Late menarcheal age [39, 40]^a^	Early menarcheal age [33, 35, 39, 43]
Early menarcheal age [19–27]	Early menarcheal age [43, 58–60]	
Never use of hormonal contraceptives [21, 28–32]		Never use of hormonal contraceptives^a^
High variability in cycle length prior to age 40 years [23]^a^		
Low number of gestations [6, 17, 19, 21–23, 25–28, 33–36]^a^	Low number of gestations [39–42]	Low number of gestations [39, 40]
Never or short period of lactation [22, 37]	Lactation length^a^	
Long period of lactation [24]		
Regular bleeding pattern before the climacterium [24, 25, 36]	Irregular bleeding pattern before the climacterium^a^	
Short or normal length of menstrual cycle in the climacterium [21, 23, 28, 38]^a^	Normal length of menstrual cycle in the climacterium^a^	
	Normal length of menstrual flow in the climacterium^a^	
	Late menopausal age [6, 25, 26, 30, 33, 35]	Late menopausal age [33, 35]
		Early age at menopause [52]
	Postmenopausal status [40, 43–51]^a^	Postmenopausal status [44–47, 51, 53–56]^a^

^a^The present study could confirm these relations

- Both early and late menarcheal age [17–27]
- Never use of hormonal contraceptives [21, 28–32]
- High variability in cycle length prior to age 40 years [23]
- Low number of gestations [6, 17, 19, 21–23, 25–28, 33–36]
- Never or short period of lactation [22, 37] but also long period of lactation [24]
- Regular bleeding pattern before the climacterium [24, 25, 36]
- Short or normal length of menstrual cycle in the climacterium [21, 23, 28, 38]

While the following predictive characteristics of reproductive life for obesity in the menopausal transition were found:

- Both early and late menarcheal age [39, 40]
- Low number of gestations [39–42]
- Lactation length
- Late menopausal age [6, 25, 26, 30, 33, 35]
- Postmenopausal status [40, 43–51]

And according to the results of the former menopause studies, the following reproductive life characteristics associated with the risk of abdominal obesity:

- Early menarcheal age [33, 35, 39, 43]
- Low number of gestations [39, 40]
- Late menopausal age [33, 35]
- Early age at menopause [52]
- Postmenopausal status [44–47, 51, 53–56]

The Cox proportional hazards regression modelling revealed that (1) the ever use of contraceptives, long menstrual cycles and higher number of gestations related to lower risk of earlier menopause, while (2) later menarcheal age, normal length of menstrual cycle or bleeding in the climacterium, irregular bleeding pattern and the postmenopausal status were associated with higher prevalence of obesity and (3) the never use of contraceptives, the regular menstruation, the postmenopausal status and relatively earlier age at menopause increased the risk of abdominal obesity in the studied sample. Overall, the characteristics of reproductive life were found to have significant influence on the onset of the menopausal transition. Moreover, the presented results suggest that the risk of obesity is also related to the reproductive life parameters.

## Conclusions

Contrary to expectations, the results from this study suggest there is no relationship between age of menarche and age at menopause. The results of the present study add to a growing body of literature showing that prior use of oral contraceptives, longer mean cycle length and smaller number of gestations are all associated with later age of menopause.

Our results also confirm those from previous studies showing that the later age of menarche, the irregular bleeding pattern before climacterium, the normal length of menstrual cycles and menstrual bleedings in the climacterium and the postmenopausal status were associated with a higher risk of obesity in climacterium.

By considering the relationship between the reproductive life characteristics and body fatness distribution in the menopausal transition, these findings confirm—beyond the never use of hormonal contraceptives—the formerly evidenced association that the menopausal status was related to abdominal obesity.

Recent studies have shown that variation in age at menopause and the other biological characteristics of the menopausal transition, e.g. body structural changes, are associated with several factors, such as genetic, reproductive, socio-demographic and certain behavioural, lifestyle influences [4, 5, 57]. These analyses confirmed the relationship between the ever use of hormonal contraceptives, the length of menstrual cycle and bleeding in the climacterium with the age of menopause and the same reproductive variables and menopausal status with the body fatness in the menopausal transition.
